# RF-Based Moisture Content Determination in Rice Using Machine Learning Techniques

**DOI:** 10.3390/s21051875

**Published:** 2021-03-08

**Authors:** Noraini Azmi, Latifah Munirah Kamarudin, Ammar Zakaria, David Lorater Ndzi, Mohd Hafiz Fazalul Rahiman, Syed Muhammad Mamduh Syed Zakaria, Latifah Mohamed

**Affiliations:** 1 Faculty of Electronic Engineering Technology, Universiti Malaysia Perlis (UniMAP), Arau 02600, Perlis, Malaysia; norainiazmi@studentmail.unimap.edu.my (N.A.); smmamduh@unimap.edu.my (S.M.M.S.Z.); 2 Advanced Sensor Technology, Centre of Exellence (CEASTech), Universiti Malaysia Perlis (UniMAP), Arau 02600, Perlis, Malaysia; ammarzakaria@unimap.edu.my (A.Z.); hafiz@unimap.edu.my (M.H.F.R.); 3 Faculty of Electrical Engineering Technology, Universiti Malaysia Perlis (UniMAP), Arau 02600, Perlis, Malaysia; latifah@unimap.edu.my; 4 School of Computing, Engineering and Physical Sciences, University of the West of Scotland, Paisley PA1 2BE, UK; David.Ndzi@uws.ac.uk

**Keywords:** moisture content measurement, neural network, smart farming, double frequency, grain moisture content, radio frequency

## Abstract

Seasonal crops require reliable storage conditions to protect the yield once harvested. For long term storage, controlling the moisture content level in grains is challenging because existing moisture measuring techniques are time-consuming and laborious as measurements are carried out manually. The measurements are carried out using a sample and moisture may be unevenly distributed inside the silo/bin. Numerous studies have been conducted to measure the moisture content in grains utilising dielectric properties. To the best of authors’ knowledge, the utilisation of low-cost wireless technology operating in the 2.4 GHz and 915 MHz ISM bands such as Wireless Sensor Network (WSN) and Radio Frequency Identification (RFID) have not been widely investigated. This study focuses on the characterisation of 2.4 GHz Radio Frequency (RF) transceivers using ZigBee Standard and 868 to 915 MHz UHF RFID transceiver for moisture content classification and prediction using Artificial Neural Network (ANN) models. The Received Signal Strength Indicator (RSSI) from the wireless transceivers is used for moisture content prediction in rice. Four samples (2 kg of rice each) were conditioned to 10%, 15%, 20%, and 25% moisture contents. The RSSI from both systems were obtained and processed. The processed data is used as input to different ANNs models such as Support Vector Machine (SVM), K-Nearest Neighbour (KNN), Random Forest, and Multi-layer Perceptron (MLP). The results show that the Random Forest method with one input feature (RSSI_WSN) provides the highest accuracy of 87% compared to the other four models. All models show more than 98% accuracy when two input features (RSSI_WSN and RSSI_TAG2) are used. Hence, Random Forest is a reliable model that can be used to predict the moisture content level in rice as it gives a high accuracy even when only one input feature is used.

## 1. Introduction

Grain (e.g., rice, wheat, corn) is the major crop and staple food source worldwide. Rice is the staple food in Asian countries and thus, is a main consideration in food security. The moisture content of grains is one of the important parameters for grain quality control especially during harvesting, milling, and storage [1]. Table 1 presents the ideal moisture contents of the different types of grain during harvest and safe storage. The moisture content of harvested paddy is usually high (19–25%) and thus needs to be dried to 14% or less for safe storage [2,3]. Grain wastage often occurs due to improper storage conditions where high moisture content promotes the growth of mould and insect infestation whereas very dry grains are brittle and susceptible to breakage. Moisture content in the grain is also affected by the weather where moisture content becomes high during the rainy season, otherwise too low in the summer or hot season. Therefore, continuous monitoring is critical especially in tropical climate where the weather varies throughout the year.

After harvesting, grains are typically stored in silos (cylindrical storage) [4]. Since silos are quite large (e.g., a diameter and height range from 4.6 m to 18.3 m and 4.6 m to 28.7 m, respectively), the moisture content may not be uniformly distributed in the silo. The moisture content measurements are not carried out frequently enough to observe the dynamics of moisture content changes within the bin continuously. Over-drying usually occurs in the bottom layer of the silo during the drying process. Therefore, a system to continuously monitor the moisture content in the storage container is important. Various methods are available for moisture content measurement as presented in Section 2. However, most of the existing methods are expensive, complex and do not provide continual automated monitoring.

**Table 1:** Moisture content during harvest and storage for a different type of grains.

Grains	Moisture Content (%)	
	Harvest	Storage
Paddy	19–25% [2,3]	<14% [2,3]
Rice		
Wheat	18–20% [5]	12.5% [5]
Maize	~40% [5]	<14% [6]

This paper is comprised of five sections: Section 1 introduces the problems that need to be overcome; Section 2 briefly describes the related fundamental knowledge and literature review of related studies; Section 3 describes the experimental method and data modelling, and Section 4 presents the results and discussion. The conclusions are presented in Section 5.

## 2. Related Works

Research studies on grain crops have been widely conducted on various aspects such as crops yield prediction [7], grain quality [8], soil moisture [9,10], and moisture content detection [11,12,13,14,15]. Numerous research studies have been conducted to find a reliable method to measure the moisture content in seasonal grain crops such as paddy, peanuts, and corn [16,17,18]. Amongst the popular methods proposed and used are dielectric method [19,20], oven drying method, and RF-based method [21]. Generally, the methods to determine the moisture content of grain can be categorized into two groups: direct and indirect methods. In the direct method, water inside the grain is removed completely. Meanwhile, the indirect method involves the measurement of the electrical properties of the grain.

The oven-drying method is one of the most accurate techniques and is widely accepted as a standard method for moisture content determination. In the oven-drying method, the sample is heated to 130 °C for 19 h [8]. However, the existing method such as microwave oven drying is expensive, destructive and time-consuming as the sample needs to be dried for long period [22]. In addition, the oven-drying method is often conducted in the laboratory because the equipment is fixed at a location due to its size and power requirement. These limitations have led to studies to develop alternative methods that are faster and cheaper. A cheaper, indirect and portable device for moisture content measurement generally called moisture metres have been developed [23,24,25]. Moisture metres operate based on the concept of dielectric constant. There are two popular types of moisture metres: the resistance-based and capacitance-based moisture metres. The moisture metre is widely used due to its low-cost, portability and is battery powered. However, more accurate moisture measurements are still carried out manually. Therefore, automatic continuous moisture content monitoring is needed to ensure safe storage of grain.

Non-destructive testing method is important especially in industries that produce high-value products [26]. As a result, research to develop a better alternative solution for moisture content determination in rice product is important. Hence, electromagnetic waves measurement is a promising method for moisture content determination. The technique involves the transmission of radio wave signals through the grain whose moisture content is to be measured. This is non-destructive and, hence, the quality of the sample can be preserved. Numerous studies have been conducted and various methods for measuring moisture content in grain have been proposed. Some studies utilised an experimental setup consisting of Vector Signal Generator (VSG), Vector Network Analyzer (VNA), and horn antennas [27,28,29,30]. In a different study, Lewis et al. constructed a system based on microwave signal operating at 5.8 GHz with two antennas, a humidity sensor, and two temperature sensors placed inside the silo [4]. Related works by Trabelsi et al. determined moisture content using instantaneous temperature [31]. Meanwhile, the system proposed in [4] monitors the moisture content as the grain is dried and the system has to be recalibrated each time the grain is added or removed. The drawback of the approach in [4] is that the calibration of the system is time-consuming because it employs the oven-drying method. Therefore, instead of using a fixed mathematical function to obtain the moisture content, a continuous intelligent monitoring system utilising machine learning could offer a better solution.

Data mining and machine learning techniques are widely used in data analysis for various objectives such as pattern identification, and decision making with minimal human intervention. Some examples of machine learning applications include human activity recognition [32], face recognition [33], traffic predictions [34,35], weather prediction [36,37], stock market prediction [38], health prediction [39], etc. Hence, some studies have integrated machine learning in their solution to determine the moisture content in grain. For example, Tahir et al. determined the moisture content through feature extraction from the digital images and moisture content classification using neural networks [40]. In a different study, Liu et al. focused on optimising the neural network topology used to predict the moisture content of grain during the drying process [41]. In addition, studies utilising a variety of classification have been conducted such as in [9] using ANN to grade the grain based on visual features. However, Bains and Kalsi used the Naïve Bayes method to predict the production of wheat [42]. Table 2 lists some of the methods and models used to determine moisture content in grains [8,22,40,43].

To the best of the authors’ knowledge, no study has been conducted using the UHF frequency band to determine the moisture content of grains. Based on previous research studies, this paper proposed a novel method of moisture content determination of grain (focusing on rice) using the combination of dual frequencies which is 2.4 GHz and 915 MHz with machine learning techniques for continuous non-destructive monitoring. Two wireless technologies have been selected which are 2.4 GHz radio frequency transceivers using the ZigBee (IEEE 802.15.14) Standard [44] and UHF RFID transceiver. The objective of this study is to investigate the effect of wireless signal transmission through rice grain (placed as an obstacle between the transmitter and receiver nodes) and use machine learning classification and prediction methods to determine the moisture content. This study characterises, compares and evaluates the robustness of wireless transmission for moisture content detection when combined with Artificial Neural Network (ANN) algorithms. Machine learning can be categorised into four major groups; supervised, unsupervised, semi-supervised, and reinforcement learning. The following are the basic concept of several ML techniques used, which includes SVM, K-Nearest Neighbor (KNN), Random Forest, and MLP.

### 2.1. Support Vector Machine

Support Vector Machine (SVM) is a well-known supervised learning algorithm that can be used for both classification and regression. SVM concept is based on the idea of finding a hyperplane that best separates a dataset into two groups. However, if the data is non-linear, the SVM algorithm has little ability to separate the hyperplanes. There are other methods available to classify non-linear data, which are known as kernel functions. Kernel functions are mathematical functions that take data as input and transform it into the required form [45]. Kernel functions mapped data to a higher dimension. There are different types of kernel functions in the SVM (linear, polynomial, Gaussian kernel, and radial basis function (RBF), sigmoid, etc.) that can be used to classify data. However, the most widely used kernel function is RBF. The hyperplane for the linear support vector machine is represented by Equation (1). Meanwhile, Equations (2)–(4) show some typical kernels for a non-linear support vector machine.

(1) Linear hyperplane, WX+b=0

(2) $$K\left(x,y\right)={\left(x\cdot y+1\right)}^{p}$$

(3) $$K(x,y)=e^{-\frac{{\left\|x-y\right\|}^{2}}{2\sigma^{2}}}$$

(4) $$K\left(x,y\right)=\tanh\left(kx\cdot y-\delta \right)$$

### 2.2. Random Forest

The basic building block of Random Forest technique is Decision Tree (DT). DTs involve a set of questions and answers to reduce the range until there is sufficient confidence to make a single prediction. Predictions made by individual DT may not be accurate, however, the combination of many DTs into a single model increases the accuracy of the prediction. The combination of multiple DTs is called Random Forest. Random Forest is a type of supervised machine learning algorithm that also can be used for both regression and classification tasks. 

### 2.3. K-Nearest Neighbour 

K-Nearest Neighbour (KNN) is a supervised machine learning algorithm that calculates the distance of a new data point to all other training data points. One of the distance functions (see Table 3) can be used to calculates the distance between the new data point with the selected K-nearest points. K represents the number of neighbours around the new data point. Table 3 listed some distance functions that can be used to calculate the distance between the new data point and its K neighbours. Based on the distance, the new data point will be classified to the class which the majority of the neighbours belong to.

### 2.4. Multilayer Perceptron 

**Figure 1:**
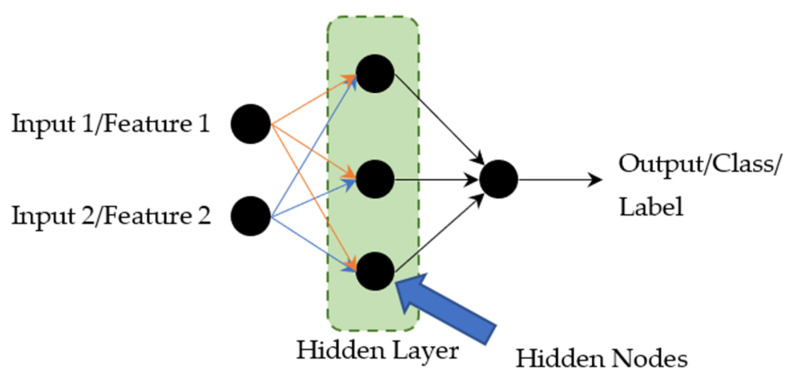
Simple Neural Network.

Multilayer Perceptron (MLP) is one of the classifier models in ANN, which is a brain-inspired computational network intended to replicate the way that the brain learns. A simple neural network consists of three layers of nodes known as the input layer, hidden layer, and output layers. A multi-layered network (having more than two layers) means that the network has at least one hidden layer (all the layers between the input and output layers are called hidden layer) as shown in Figure 1.

## 3. Experimental Measurement

**Figure 2:**
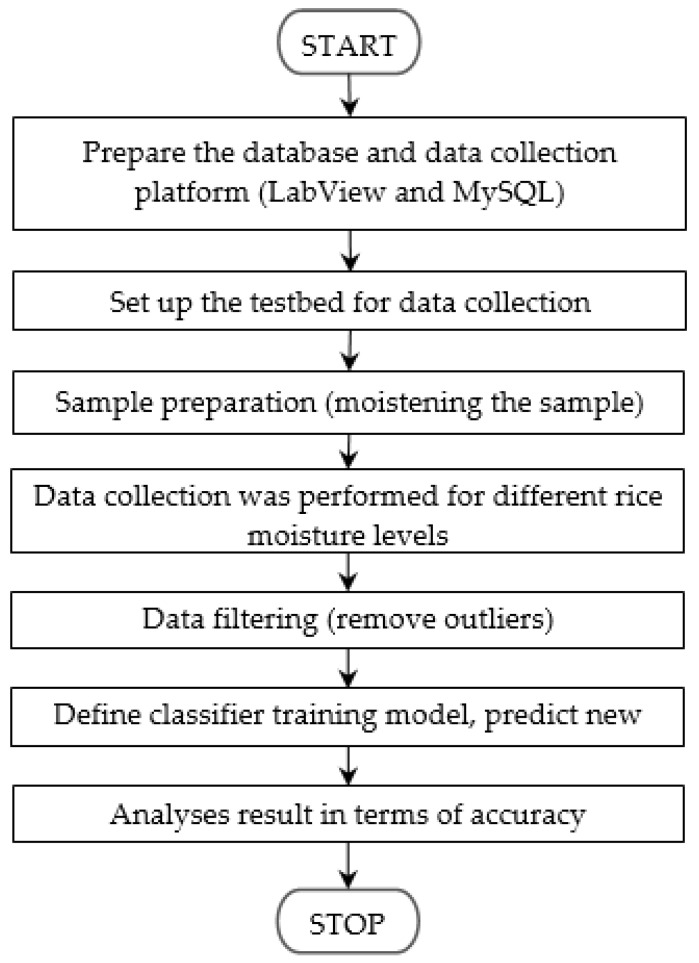
Research flow.

In the authors’ previous work [46], wireless technology (WiFi) operating in the 2.4 GHz band was utilized. In this paper, low cost RFID technology operating in the 868 MHz to 915 MHz frequency band and Zigbee (IEEE802.15.4) based WSN operating in 2.4 GHz band are proposed to improve the performance of the previous method. Figure 2 shows the flow diagram of this research.

### 3.1. Sample Preparation

The moisture content in the rice was raised to the desired levels using distilled water. Moistening using distilled water is a common technique that has been used by some researches [47,48,49,50,51,52]. Some researchers calculate the quantity of the water required; where the moisture content is in terms of wet basis (w.b.) [2,8,48,49,50], while other studies used dry basis (d.b.) [47,51,52]. The amount of distilled water (*Q*) in kg required to achieve a certain moisture content can be calculated using Equation (5).

(5) $$Q=\frac{W_{i}(M_{d}-M_{i})}{\left(100-M_{d}\right)}$$

where *W_i_* is the initial mass of the sample in kg, *M_i_* is the initial moisture content of sample as % w.b., *M_d_* is the desired moisture content of the sample in % w.b., and *Q* is the mass of water to be added in kg. 

To condition the rice to the desired moisture content, the following process was performed: (1) the initial moisture content for each bag was measured using a commercial moisture metre; (2) the weight of rice in the polyethene bag was measured; (3) the amount of distilled water (*Q*) required to moisten the rice was calculated; (4) the required amount of distilled water was then added to each bag of rice; (5) the polythene bags were resealed. The samples were then kept in a refrigerator at 4 °C for 72 h to ensure uniform moisture distribution. After 72 h, the rice was taken out from the refrigerator and allowed to warm up to room temperature for 2 h.

The samples were prepared using the method described in a previous paper [43]. Each sealed polythene bag contained 2 kg of rice. Four bags of rice with different moisture levels of 10%, 15%, 20%, and 25% were prepared. The samples were labelled A, B, C, D and E for range 0% (no sample), 9–11%, 14–16%, 19–21%, and 24–26%, respectively, as shown in Table 4.

### 3.2. Experimental Setup and Data Collection

Radio wave signals that travel from the transmitter to the receiver will suffer attenuation due to a variety of different phenomena such as multipath, reflection, scattering, refraction, diffraction, and absorption [53]. For all measurements, the setup and location were the same for all samples. To maximise the signal strength and maintain a good connection between the transmitter and the receiver, the general rule of thumb is that the first Fresnel zone must be 60% clear of obstruction from the centre line of sight to the outer boundary of the first Fresnel zone. Since the distance between the TX and RX was set to 50 cm, both the TX and RX should be placed at a height of approximately 12 cm.

**Figure 3:**
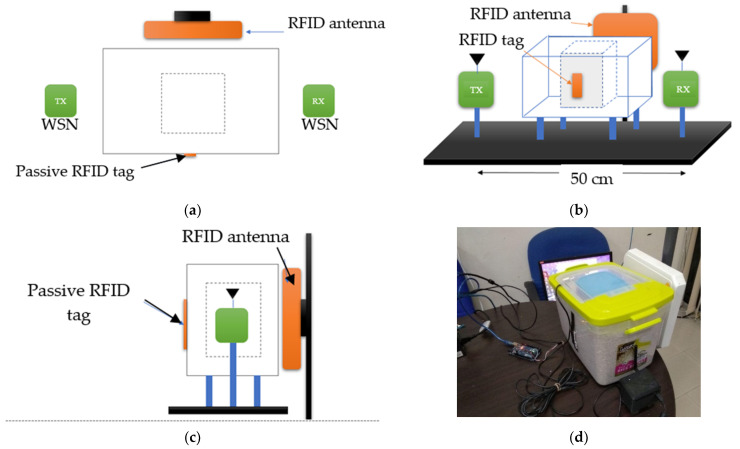
The experimental setup considering the Fresnel zone clearance: (**a**) the top view; (**b**) front view; (**c**) the side view of the experimental testbed layout; (**d**) a picture of the setup.

The experimental tests were conducted in an environment without interference from other wireless systems. The experimental setup used is shown in Figure 3. Figure 3a–c show the top, front and side views of the setup, respectively. Figure 3d is the picture of the actual experimental setup.

The experimental setup comprises of two wireless sensor nodes, one passive RFID tag, one RFID antenna, 7-litre container, 3-litre (small) container, PVC pipe as a stand for the wireless sensor nodes and the storage box. The 3-litre (small) container was placed in the middle of the 7-litre container. The 3-litre container contained the sample with different moisture levels. A set of transmitter and receiver used consists of a development board known as Waspmote. Each Waspmote board was attached to an XBee Series 1 transceiver. The XBee Series 1 operates based on IEEE 802.15.4 standard in the 2.4 GHz frequency band. The RFID system used includes two passive RFID tags (see Table 5) and an RFID reader from ThingsMagic-m6e [54]. For easier identification during data collection, the ALN-9654 tag is labelled as RFID_TAG1 while PMT-06W tag is labelled as RFID_TAG2. The reader has 4 available ports for antenna connections. However, in this study, only 1 port was connected to a directional antenna. The RSSI data from the RFID tags are stored in a database. The RSSI data are collected through the serial communication port connected to a personal computer (PC). A graphical user interface was developed using LabView to facilitate the data collection process. Data from the RFID and WSN were then captured and synchronized based on date and time and saved. The total data collected from the experimental tests is 10,000 samples such that each moisture content label (0%, 10%, 15%, 20% and 25%) comprised of 2000 data samples.

**Figure 4:**
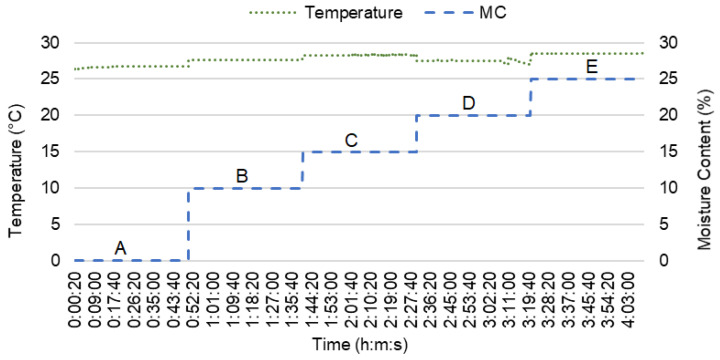
The temperature during testing.

Throughout the experimental tests, the temperature range was between 26.38 °C to 28.94 °C. The temperature readings recorded during the experimental test is shown in Figure 4. 

### 3.3. Model Design and Training

**Figure 5:**
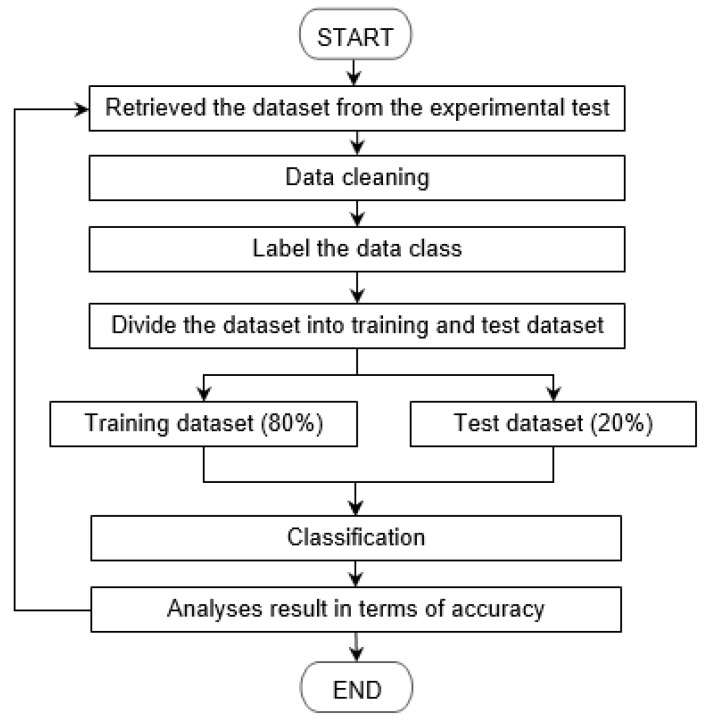
Classification process.

Analysis and modelling were done in Python using the Scikit-learn library and Google Collaboratory (online application for deep learning), which provides free access to Goggle General Processing Unit that reduces the process training time. Amongst the models used are SVM, Random Forest, KNN, and MLP. Figure 5 shows the flow of the classification process. 

The collected data were pre-processed before applying any classification or clustering technique. As part of pre-processing, the data was plotted using a box plot where any outliers were identified and replaced with the average values. The data is divided into training, validation, and testing sets. A common approach is for a dataset to be split into 80% for training and 20% for validation. The data was then fed into a different machine learning model.

### 3.4. Model Performance Analysis

Various measures that can be used to assess the performance of a model. These measures include the determination coefficient (*R*^2^), Mean Square Error (MSE), Mean Absolute Error (MAE) and Root Mean Square Error (RMSE) [34]. The *R*^2^, MAE, MSE and RMSE can be calculated using Equations (6)–(9), respectively.

(6) $$\mathrm{Determination}\;\mathrm{of}\;\mathrm{coefficient},\;R^{2}=\frac{\sum_{i=1}^{n}{\left(t_{i}-p_{i}\right)}^{2}}{\sum_{i=1}^{n}t_{i}^{2}\sum_{i=1}^{n}p_{i}^{2}}$$

(7) $$\mathrm{Mean}\;\mathrm{Absolute}\;\mathrm{Error}\;(\mathrm{MAE})\;=\frac{1}{n}\sum_{i=1}^{n}\left|t_{i}-p_{i}\right|$$

(8) $$\mathrm{Mean}\;\mathrm{Square}\;\mathrm{Error}\;(\mathrm{MSE})\;=\frac{1}{n}\sum_{i=1}^{n}{\left(t_{i}-p_{i}\right)}^{2}$$

(9) $$\mathrm{Root}\;\mathrm{Mean}\;\mathrm{Square}\;\mathrm{Error}\;(\mathrm{RMSE})\;=\sqrt{\frac{1}{n}\sum_{i=1}^{n}{\left(t_{i}-p_{i}\right)}^{2}}$$

where *n* is the total number of samples and, $t_{i}$ and $p_{i}$ are the measured (true value) and predicted values for (*i* = 1,2,3,…,*n*), respectively. The range *R*^2^ is (0, 1) where the performance of the model is best when *R*^2^ = 1.

A confusion matrix is a technique used for summarising the performance of a classification algorithm and it can be plotted to better understand and compare different classification techniques. There are a few measures that can be derived from the confusion matrix including accuracy, precision, F-score, and recall. True positive (*TP*) is when the outcome predicted is positive and it is true, true negative (*TN*) is when the outcome predicted is negative and it is true, false positive (*FP*) is when the outcome predicted is positive and it is false and false negative (*FN*) is when the outcome predicted is negative and it is false.

- Accuracy (*ACC*) is calculated as the number of all correct predictions divided by the total number of a sample dataset. The best *ACC* is 1.0, whereas the worst is 0.0. It can also be calculated by 1—ERR.

(10) $$ACC=\frac{TP+TN}{TP+TN+FN+FP}$$

- Sensitivity (also known as Recall or true positive rate) is calculated as the number of correct positive predictions divided by the total number of positives. The best Recall is 1.0, whereas the worst is 0.0.

(11) $$REC\;\mathrm{or}\;SN=\frac{TP}{TP+FN}$$

- Precision (positive predictive value) is calculated as the number of correct positive predictions divided by the total number of positive predictions. It is also called positive predictive value (PPV). The best precision is 1.0, whereas the worst is 0.0.

(12) $$PREC=\frac{TP}{TP+FP}$$

- F-score is a harmonic mean of precision and recall.

(13) $$F_{\beta }=\frac{\left(1+\beta^{2}\right)\left(PREC\cdot REC\right)}{\left(\beta^{2}\cdot PREC+REC\right)}$$

where *β* is commonly 0.5, 1, or 2.

The *β* parameter determines the weight of recall and precision in the F-score. When *β* is 0.5, more weight on precision, less weight on recall. When *β* is 1, weight is balanced between precision and recall. Lastly, when *β* is 2, less weight on precision, more weight on recall.

## 4. Results and Discussion

The data was collected in an indoor laboratory environment using WSN and RFID. The RSSI from both wireless devices after the signal pass through the samples with different moisture contents were measured.

**Figure 6:**
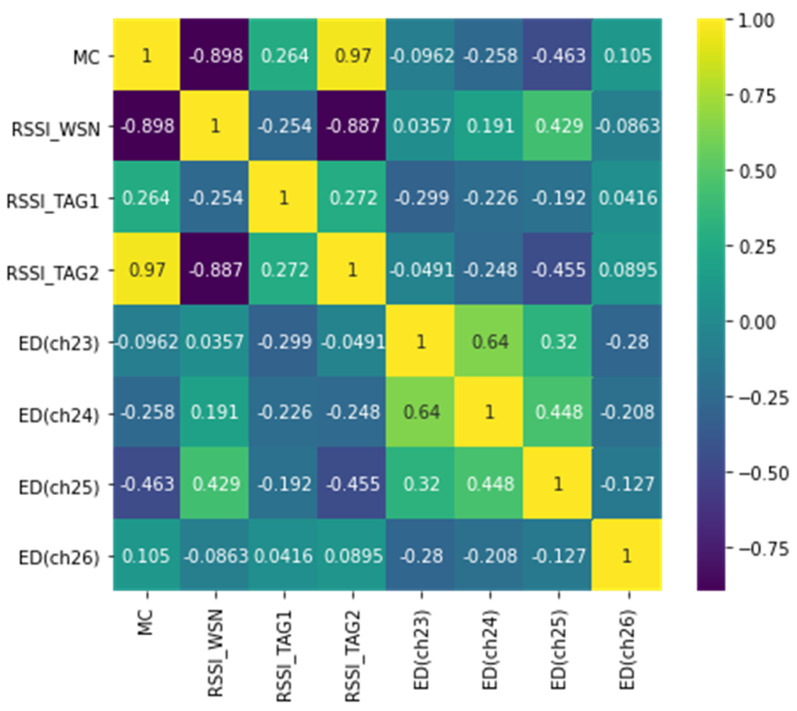
Correlation between different variables. MC represent Moisture Content; ED represents Energy Detection.

The correlation between different variables is shown in Figure 6. From the correlation plot, moisture content has a positive relationship with TAG1 and TAG2, where the correlation value is 0.264 and 0.970, respectively. In contrast, the RSSI_WSN has a negative relationship with moisture content with a correlation value of −0.898. Therefore, from the correlation plot, the RSSI_WSN, RSSI_TAG1, and RSSI_TAG2 are significant compared to other variables and will be used in the classification and prediction of moisture content in rice.

**Figure 7:**
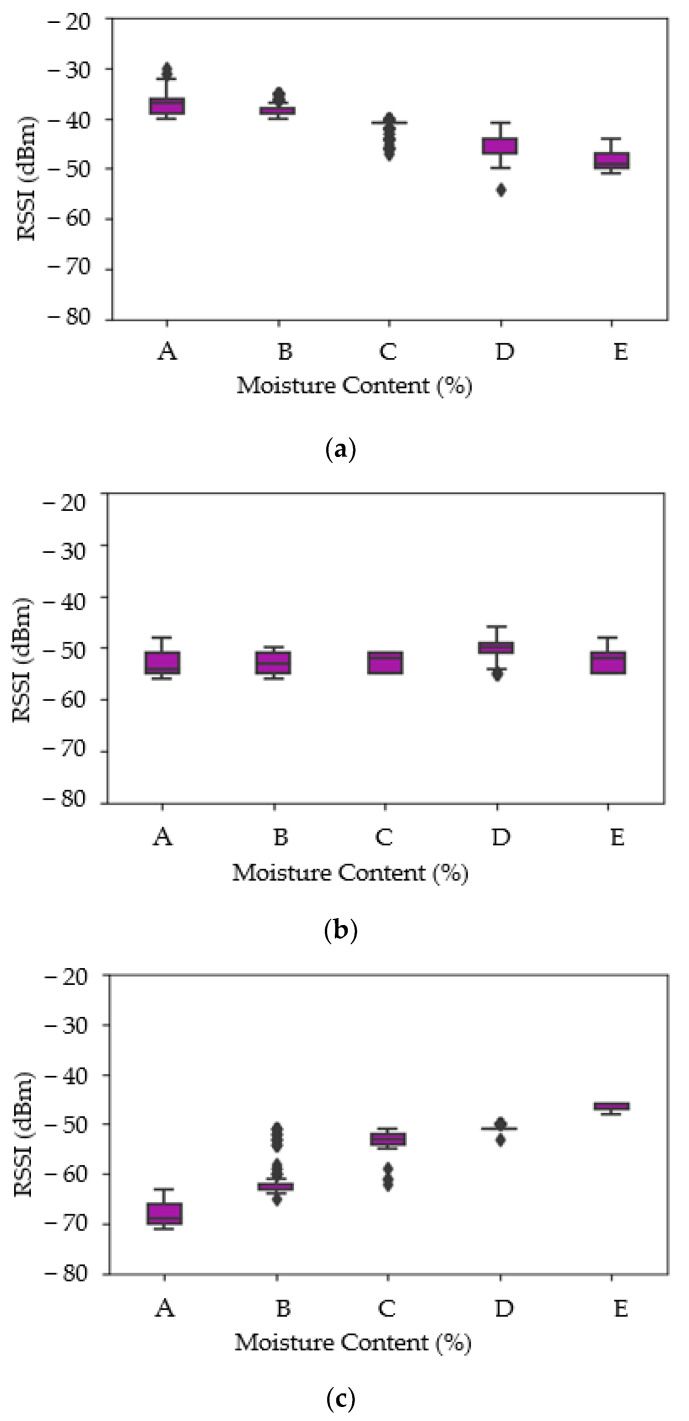
Boxplot of RSSI of WSN vs. MC, RSSI of TAG1 and MC, and RSSI of TAG2 and MC. (**a**) RSSI of WSN vs. MC. (**b**) RSSI of TAG1 vs. MC. (**c**) RSSI of TAG2 vs. MC.

Boxplots of each feature are shown in Figure 7, which help to determine outliers, range, mean, median, minimum, and maximum values. Figure 7a shows the RSSI measured from WSN versus moisture content. Figure 6c and Figure 7b present results from two different types of RFID tags (named as TAG1 and TAG2) versus moisture content, respectively. The RSSI plotted for WSN shows a decreasing pattern with increasing moisture content of rice. Conversely, the RSSI values from TAG2 show an increasing pattern with increasing moisture content. The difference in signal strength trend can be attributed to the wavelength of the signal relative to the test sample bag and its contents. The moisture is thought to enhance surface waves, hence enhancing signal strength at certain frequencies whilst increasing signal attenuation at others, especially higher frequencies [55]. Unlike WSN and TAG2, there is no clear pattern that can be observed from TAG1 and this indicates that there are no differences in the RSSI reading for different moisture content levels in the samples. Furthermore, a negative pattern can be observed in Figure 7a whilst Figure 7c shows a positive pattern.

**Figure 8:**
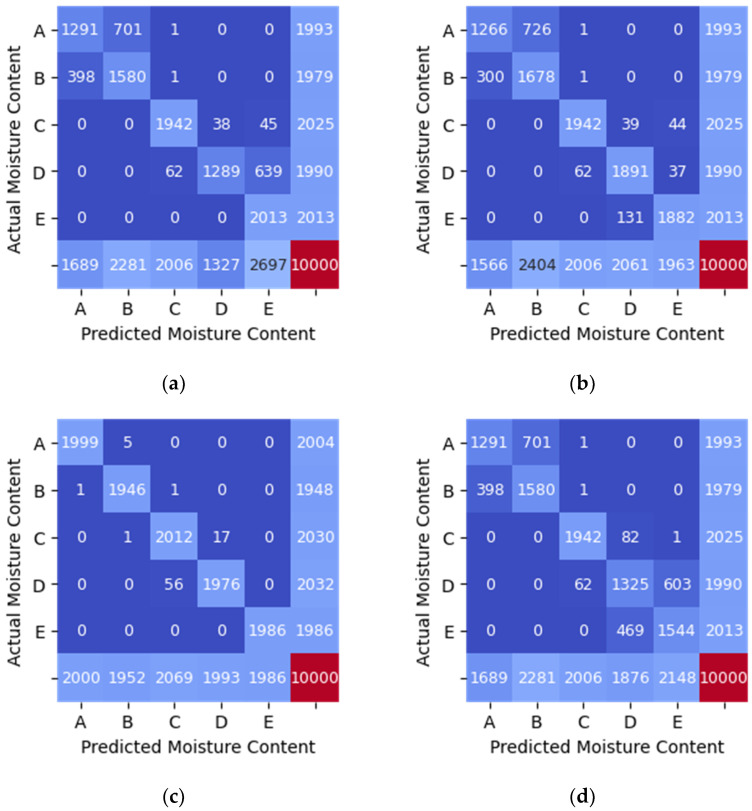
Confusion Matrix Heatmap of RSSI_WSN data. (**a**) SVM (linear kernel). (**b**) Random Forest. (**c**) KNN (*n* = 5). (**d**) MLP.

**Figure 9:**
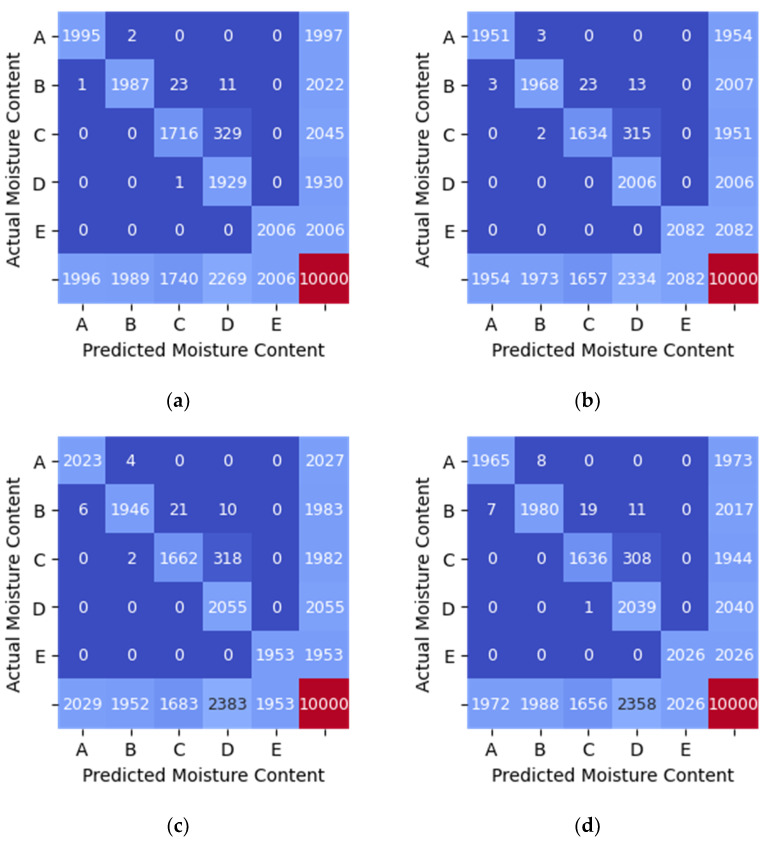
Confusion Matrix Heatmap of RSSI_TAG2 data. (**a**) SVM (linear kernel). (**b**) Random Forest. (**c**) KNN (*n* = 5). (**d**) MLP.

**Figure 10:**
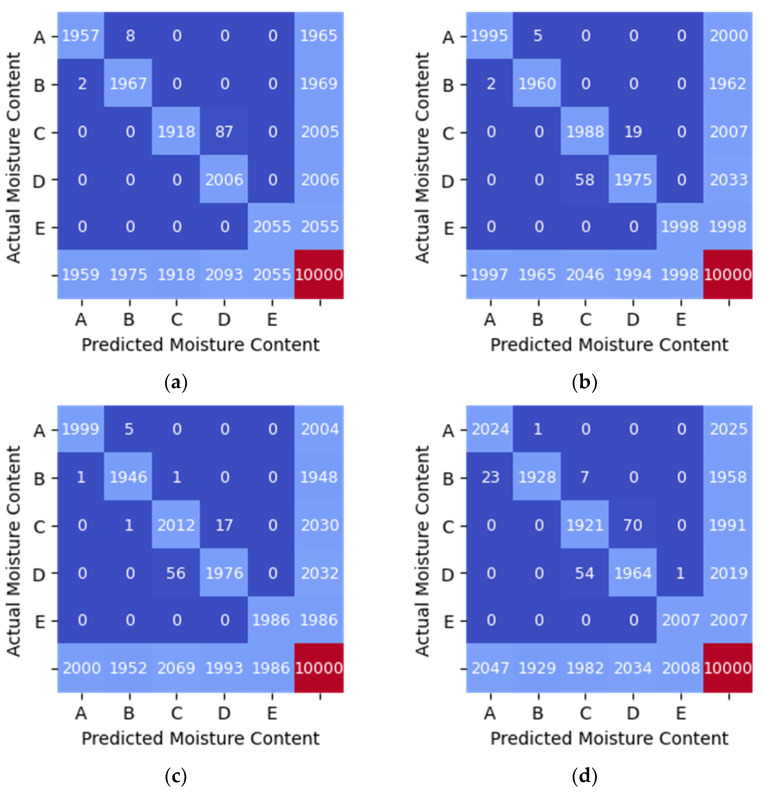
Confusion matrix heatmap of different classification method using 2 features (RSSI_WSN and RSSI_TAG2). (**a**) SVM (linear kernel). (**b**) Random Forest. (**c**) KNN (*n* = 5). (**d**) MLP.

The performance of each model is depicted in Table 6, Table 7 and Table 8. Additionally, the confusion matrices are shown graphically as heatmaps from Figure 8, Figure 9 and Figure 10. Based on the confusion matrix heatmap, the x-axis represents the predicted moisture content using the algorithms while the y-axis is the actual moisture content (label) of the sample (rice). The elements in the diagonal are the correctly classified moisture contents, while the rest of the elements are misclassified.

The classification of moisture content when the RSSI from WSN was used as features (input) to the four machine learning algorithms indicates that Random Forest has the highest accuracy of 0.87 as in Table 6. On the other hand, MLP shows the lowest classification accuracy of 0.77. The values in the diagonal elements in the confusion matrix should be 2000 for each moisture content range if the model can predict with 100% accuracy. The value in the diagonal elements is either lower or higher than 2000 due to misclassification. For example, in Figure 8a, the value in the element to predict moisture content for sample A is 1291, indicating that the model can correctly predict 1292 actual moisture content, while 701 and 1 were wrongly predicted as the moisture content in the range of sample B and C, respectively.

In the classification of moisture content when the RSSI data from TAG2 was used as input to the four machine learning algorithms, all models gave a high performance with an accuracy of 0.96 (refer Table 7). Figure 9 shows the heatmap generated from the confusion matrix for RSSI_TAG2 data. The values in the diagonal elements in the confusion matrix should be 2000 for each moisture content range if the model can predict with 100% accuracy, precision and recall.

In Figure 8 and Figure 9, only one feature was used as input to each model at a time. Whereas, Table 8 and Figure 10 show the result when two features (RSSI_WSN and RSSI_TAG2) were used as inputs to each model. All models give higher accuracy of 0.99 when the two features were used except MLP. The accuracy of the MLP model is 0.98, which is 0.01 lower than other models.

Table 9 indicated that the KNN is not suitable for classifying the moisture content as the MAE, MSE and RMSE values are high with the MAE for KNN (RSSI_WSN) and KNN (RFID_TAG1) being the highest at 9.70. From the three features (RSSI_WSN, RSSI_TAG1 and RSSI_TAG2), RSSI_TAG1 data is not suitable for determining the moisture content of rice. The SVM, Random Forest and MLP can be used to classify the moisture content. However, this study found that, based on the MAE, MSE and RMSE, the Random Forest model using the two features (RSSI_WSN and RSSI_TAG2) is the best classification model where the MAE, MSE and RMSE are 0.05, 0.28, and 0.52, respectively.

## 5. Conclusions

Based on Table 6, Table 7 and Table 8 and Figure 8, Figure 9 and Figure 10, the Random Forest model gives the highest accuracy of approximately 87% and 99% for one feature (RSSI_WSN) and two features (RSSI_WSN and RSSI_TAG2), respectively. The comparison of measures for different model indicates that the Random Forest with two features which are RSSI from WSN and RSSI from RFID’s tag (TAG2) is the best prediction model that can be used to predict the moisture content level in rice. RSSI data from WSN and TAG2 is suitable for the classification of moisture content in rice because the moisture content has a positive relationship with TAG2, where the correlation value is 0.970. Similarly, the RSSI_WSN also has a strong correlation with moisture content with a correlation value of −0.898. The results suggest that the wireless transmission (RFID system operating in the 915 MHz range and the WSN operating in the 2.4 GHz) can be used to measure the moisture content of rice. However, further study needs to be conducted to further improve the monitoring system and investigate the system under different conditions. In addition, the parameters for MLP such as the number of hidden layers, the number of iterations, different activation function can be further investigated.

## Figures and Tables

**Table 2:** Related Studies.

Study	Method	Materials	Software	Wireless Band	Analytic/Model
[40]	Machine vision technology (MVT)	Cereal grain	Pixelink Capture to capture image	Not mentioned	Four layers back propagation neural network (BPNN) model
[22]	VNA and horn antennas	Wheat	Not mentioned	10–18 GHz	Artificial neural network (ANN)
[8]	Visual classification of good or bug-damaged wheat grain	Wheat grain	WEKA, MATLAB R014b	Not mentioned	ANN, DT, DA
[43]	Dielectric measurement using free-space transmission using VNA and horn antennas	Wood	Not mentioned	8–12 GHz	Root-mean-squared error (RMSE)

**Table 3:** Distance Functions.

Functions	Formula
Euclidean	$\sqrt{\sum_{i=1}^{k}{\left(x_{i}-y_{i}\right)}^{2}}$
Manhattan	$\sum_{i=1}^{k}\left|x_{i}-y_{i}\right|$
Minkowski	${\left(\sum_{i=1}^{k}{\left(\left|x_{i}-y_{i}\right|\right)}^{q}\right)}^{1/q}$

**Table 4:** Labels used for moisture content ranges.

x-Axis Label	Moisture Range (%)	Average Moisture Content (%)
A	Empty container (as baseline)	0
B	9–11	10
C	14–16	15
D	19–21	20
E	24–26	25

**Table 5:** RFID tags specification.

Label	Brand and Image	Specifications
RFID_TAG1	ALN-9654 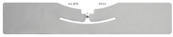	Operating in 840–960 MHz IC chip: Alien Higgs-3 Dimension: 9.65 × 2.32 cm^2^ (D × W) Communication protocol: EPCglobal UHF Class 1 Gen 2 (ISO 18000-6C) Application surface materials: Wood, plastic, glass, cardboard
RFID_TAG2	PMT-06W 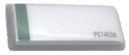	Operating at 915–920 MHz IC chip: Impinj Monza R6-P Dimension: 5.5 × 2.3 × 0.53 cm^3^ (D × W × H) Communication protocol: EPC global Class1 Gen2 compliant (ISO/IEC18000-63) Application surface materials: Wood, plastic, glass, cardboard, metal

**Table 6:** Performance of different algorithms for RSSI_WSN data.

Classification or Clustering Method	Weighted Average			
	Accuracy	Precision	Recall	F1-Score
SVM (linear kernel)	0.81	0.83	0.81	0.81
Random Forest	0.87	0.87	0.87	0.86
KNN (*n* = 5)	0.86	0.87	0.86	0.86
MLP, hidden layer (150,100,50), activation (relu), solver (adam)	0.77	0.77	0.77	0.77

**Table 7:** Performance of different algorithms for RSSI_TAG2 data.

Classification or Clustering Method	Weighted Average			
	Accuracy	Precision	Recall	F1-Score
SVM (linear kernel)	0.96	0.97	0.96	0.96
Random Forest	0.96	0.97	0.96	0.96
KNN (*n* = 5)	0.96	0.97	0.96	0.96
MLP, hidden layer (150,100,50), activation (relu), solver (adam)	0.96	0.97	0.96	0.96

**Table 8:** Performance of different classification method using 2 features (RSSI_WSN and RSSI_TAG2).

Classification or Clustering Method	Weighted Average			
	Accuracy	Precision	Recall	F1-Score
SVM (linear kernel)	0.99	0.99	0.99	0.99
Random Forest	0.99	0.99	0.99	0.99
k-NN (*n* = 5)	0.99	0.99	0.99	0.99
MLP, hidden layer (150,100,50), activation (relu), solver (adam)	0.98	0.98	0.98	0.98

**Table 9:** MAE and RMSE for different models.

Model	MAE	MSE	RMSE	Model	MAE	MSE	RMSE
SVM (RSSI_WSN)	1.46	12.71	3.57	KNN (RSSI_WSN)	9.70	150.87	12.28
SVM (RSSI_TAG1)	9.63	174.18	13.20	KNN (RSSI_TAG1)	9.70	149.85	12.24
SVM (RSSI_TAG2)	0.19	1.11	1.05	KNN (RSSI_TAG2)	9.57	147.04	12.13
SVM (RSSI_WSN, RSSI_TAG2)	0.05	0.31	0.55	KNN (RSSI_WSN, RSSI_TAG2)	9.60	148.00	12.17
Random Forest (RSSI_WSN)	1.17	11.01	3.31	MLP (RSSI_WSN)	1.55	12.60	3.55
Random Forest (RSSI_TAG1)	8.53	146.16	12.09	MLP (RSSI_TAG1)	8.28	128.77	11.35
Random Forest (RSSI_TAG2)	0.20	1.14	1.07	MLP (RSSI_TAG2)	0.25	1.67	1.30
Random Forest (RSSI_WSN, RSSI_TAG2)	0.05	0.28	0.52	MLP (RSSI_WSN, RSSI_TAG2)	0.07	0.40	0.63

## Data Availability

All data generated or appeared in this study are available upon request by contact with the corresponding author. Furthermore, the models and code used during the study cannot be shared at this time as the data also forms part of an ongoing study.
